# Candidate effector proteins from the oomycetes *Plasmopara viticola* and *Phytophthora parasitica* share similar predicted structures and induce cell death in *Nicotiana* species

**DOI:** 10.1371/journal.pone.0278778

**Published:** 2022-12-02

**Authors:** Maud Combier, Edouard Evangelisti, Marie-Christine Piron, Sebastian Schornack, Pere Mestre

**Affiliations:** 1 SVQV, UMR-A 1131, Université de Strasbourg, INRAE, Colmar, France; 2 Sainsbury Laboratory (SLCU), University of Cambridge, Cambridge, United Kingdom; University of Nebraska-Lincoln, UNITED STATES

## Abstract

Effector proteins secreted by plant pathogens are essential for infection. Cytoplasmic RXLR effectors from oomycetes are characterized by the presence of RXLR and EER motifs that are frequently linked to WY- and/or LWY-domains, folds that are exclusive to this effector family. A related family of secreted candidate effector proteins, carrying WY-domains and the EER motif but lacking the canonical RXLR motif, has recently been described in oomycetes and is mainly found in downy mildew pathogens. *Plasmopara viticola* is an obligate biotrophic oomycete causing grapevine downy mildew. Here we describe a conserved *Pl*. *viticola* secreted candidate non-RXLR effector protein with cell death-inducing activity in *Nicotiana* species. A similar RXLR effector candidate from the broad host range oomycete pathogen *Phytophthora parasitica* also induces cell death in *Nicotiana*. Through comparative tertiary structure modelling, we reveal that both proteins are predicted to carry WY- and LWY-domains. Our work supports the presence of LWY-domains in non-RXLR effectors and suggests that effector candidates with similar domain architecture may exert similar activities.

## Introduction

Oomycete plant pathogens rely on effector proteins to infect host plants and complete their life cycle. To facilitate infection, effectors typically modify host metabolism and suppress plant defenses [1], either directly in the extracellular space (apoplastic effectors) or after translocation into plant cells (cytoplasmic effectors). RXLR proteins constitute the largest and best-studied oomycete effector family. They comprise an N-terminal signal peptide, followed by RXLR and EER motifs, as well as, in many cases, one or more WY-domains. The latter are known to adopt a structural fold found only in this family of proteins [2]. Another fold, the LWY-domain, often occurs in tandem repeats, provides structural and functional modularity to RXLR effectors, and may facilitate their evolution [3].

Advances in sequencing technologies have accelerated genome sequencing of several plant-pathogenic oomycetes, which has in turn led to the discovery of their effector repertoires [4]. The last 15 years have witnessed important work devoted to the functional analysis of RXLR effectors involved in various plant-oomycete interactions, resulting in the identification of their targets and the characterization of their role as defense suppressors [5, 6]. In parallel, several authors have identified RXLR effectors that induce cell death in *Nicotiana* species [7–12], however, because in most cases cell death resulted from constitutive expression of effector proteins in a model plant, its biological significance is not understood. Indeed, the expression of effectors in non-native hosts and at levels much higher than those attained upon natural infection could lead to a cell death induction that, as it has been hypothesized, would not illustrate the actual effector function but would rather be the consequence of excessive effector activity [13].

The oomycete effector repertoire has recently been expanded with the description of secreted proteins carrying WY-domains and the EER motif, but lacking an RXLR motif [14–17]. These candidate effector proteins are mainly found in downy mildew pathogens. Their recognition by intracellular NLR disease resistance proteins suggests they fulfil their function in the host cytoplasm [17]. The fact that effectors are targeted by disease resistance proteins has been exploited to use effectors as tools to accelerate disease resistance gene discovery [18–20]. Because resistance genes are central to breeding programs, a complete knowledge of the effector repertoire of a plant pathogen is important to increase the efficiency of breeding for plant disease resistance.

The obligate biotroph *Plasmopara viticola* causes downy mildew of grapevine. This oomycete was introduced to Europe from the United States in the late 19th century and has spread worldwide [21]. The development of genomic and transcriptomic resources for *Pl*. *viticola* has enabled the candidate effector repertoire of several *Pl*. *viticola* isolates to be predicted [22–26]. Functional analysis studies then unveiled the role of some of these effector proteins in plant defense response suppression [27–30], and identified some of their host targets [31–33]. Frequently, the induction of plant cell death in *N*. *benthamiana* has been described for RXLR effectors from *Pl*. *viticola* [16, 28, 30, 34, 35].

Recently, we reported the identification of *Pl*. *viticola* candidate effector proteins containing WY-domains and EER motifs but lacking the RXLR motif [16]. Understanding the function of this class of effectors might be important to devise strategies to control grapevine downy mildew. To this end, we describe the characterization of a secreted, WY/LWY-domain containing *Pl*. *viticola* candidate effector protein, termed Pvit47, with cell death-inducing activity in *Nicotiana* species.

## Results

### Pvit47 induces cell death in *Nicotiana* species

**Fig 1:**
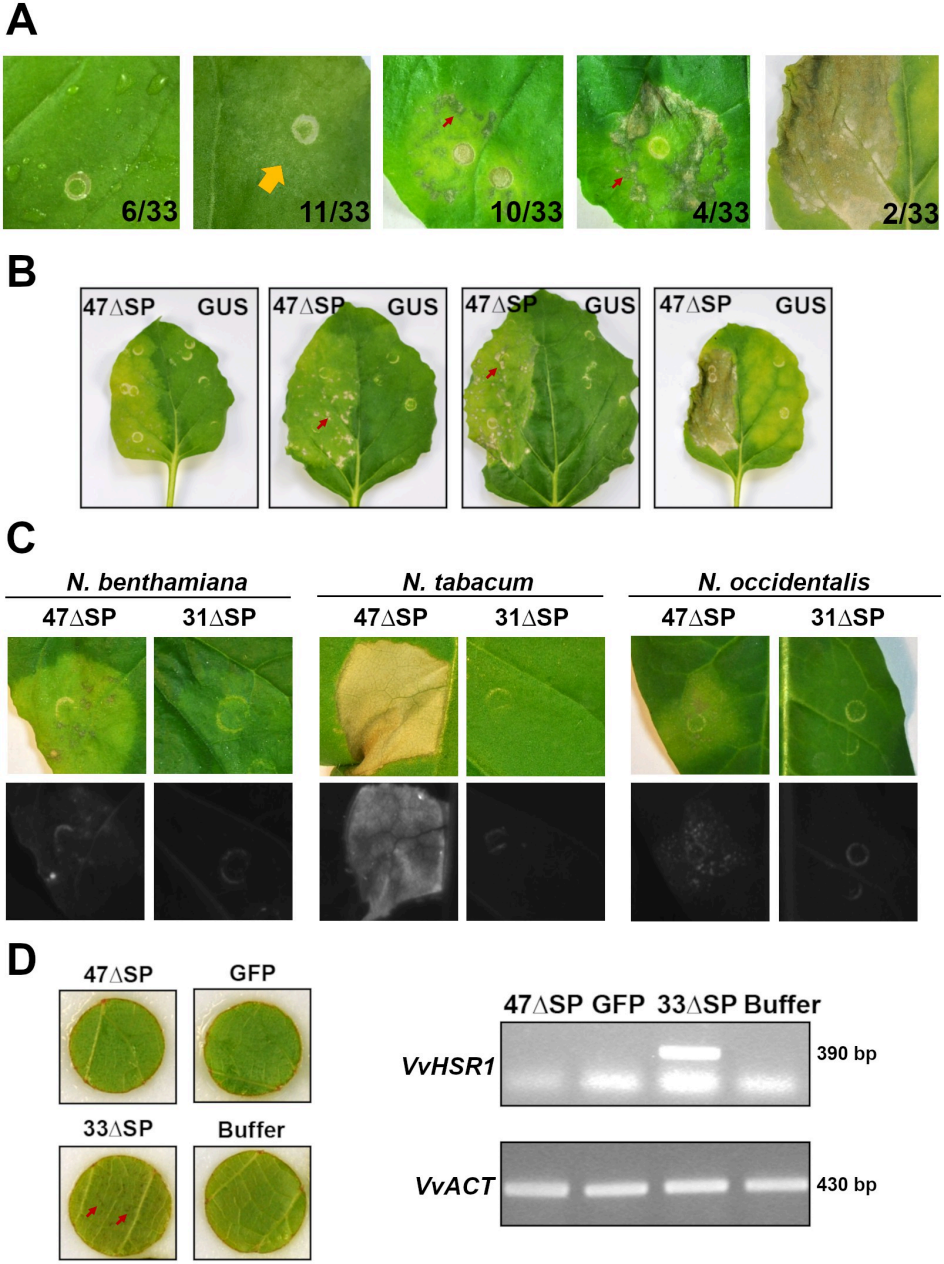
Candidate effector Pvit47 induces cell death in *Nicotiana* species. **(A)** Cell death induction following *Agrobacterium*-mediated transient expression of Pvit47ΔSP (47ΔSP) in *N*. *benthamiana* leaves. Cell death responses were assigned to one of five classes based on strength, from no response to uniform tissue collapse. Images are representative of the five classes and numbers indicate the number of responses falling inside each class. Results correspond to five independent experiments. Details of class definition and results for independent experiments are presented in S1 Table. Orange arrow indicates weak cell death and red arrows indicate patches of collapsing tissue. **(B)** Cell death induction following *Agrobacterium*-mediated transient expression of 47ΔSP in plants belonging to the same batch. *Agrobacterium*-mediated transient expression of ß-glucuronidase (GUS) was used as negative control. Arrows indicate patches of collapsing tissue. **(C)** Cell death induction following *Agrobacterium*-mediated transient expression of 47ΔSP in *Nicotiana* species. Top: daylight, bottom: blue light. *Agrobacterium*-mediated transient expression of candidate effector protein 31ΔSP, reported not to induce cell death in *Nicotiana spp*. [16], is shown for comparison. **(D)** Cell death induction following *Agrobacterium*-mediated transient expression of 47ΔSP in grapevine leaf discs. Cell death was assessed visually and as expression of the cell death marker *VvHSR1* by semiquantitative reverse transcription-polymerase chain reaction (RT-PCR). Leaf discs transiently expressing the green fluorescent protein (GFP) or infiltrated with agroinfiltration buffer are used as negative controls, while leaf discs infiltrated with *Agrobacterium* carrying the 33ΔSP construct are used as positive control. Arrows indicate cell death spots in leaf discs infiltrated with 33ΔSP. Actin (*VvACT*) is used as standard for RT-PCRs. *Nicotiana* pictures were taken at 5 days post-agroinfiltration (dpa). Grapevine pictures and sampling for RT-PCR experiments were done at 5 dpa. Each RT-PCR sample corresponds to four pooled leaf discs. Experiments were repeated two more times with the same results.

Pvit47 was identified in a previous study as a putatively secreted WY-domain-containing protein from *Plasmopara viticola* (Plvit221r1_S0324g36070) [16]. While screening candidate effector proteins for their ability to suppress cell death in *N*. *benthamiana*, we observed that *Agrobacterium*-mediated transient expression of Pvit47 lacking its signal peptide (Pvit47ΔSP) triggered cell death, which occurred at 5 days post-agroinfiltration (dpa). Cell death appearance varied from yellowing to different levels of tissue collapse (Fig 1A, S1 Table). By contrast, cell death was not observed following *Agrobacterium*-mediated transient expression of ß-glucuronidase (GUS) (Fig 1B). A range of phenotypic responses could be observed in plants from the same batch, suggesting that minor differences in leaf physiology could interfere with the extent of cell death development (Fig 1B). Expression of Pvit47ΔSP also triggered cell death in *N*. *occidentalis* and *N*. *tabacum* (Fig 1C). The response was much stronger in the latter and visible at 2 dpa. In contrast to Pvit33 [16], Pvit47ΔSP did not trigger visible cell death in grapevine and the cell death marker gene *VvHSR1* [36] was not induced (Fig 1D). Our results show that Pvit47 induces cell death in *Nicotiana* species but not in grapevine.

### Pvit47 is conserved in *Pl*. *viticola* isolates, expressed upon infection and localizes to the plant endoplasmic reticulum

To gain insight into the variability of *Pvit47*, we analyzed resequencing data [37] from 18 European isolates of *Pl*. *viticola*. *Pvit47* occurred in all of them with low nucleotide sequence variability (1.3%, 15 out of 1083 positions) and low (2.5%, 9 out of 360 residues) amino acid sequence variability (S1 Fig and S2 Table).

Consistent with transcriptomic analyses [16], *Pvit47* was expressed in isolate Pv221 in sporangia, germinated spores and during infection (S2 Fig).

**Fig 2:**
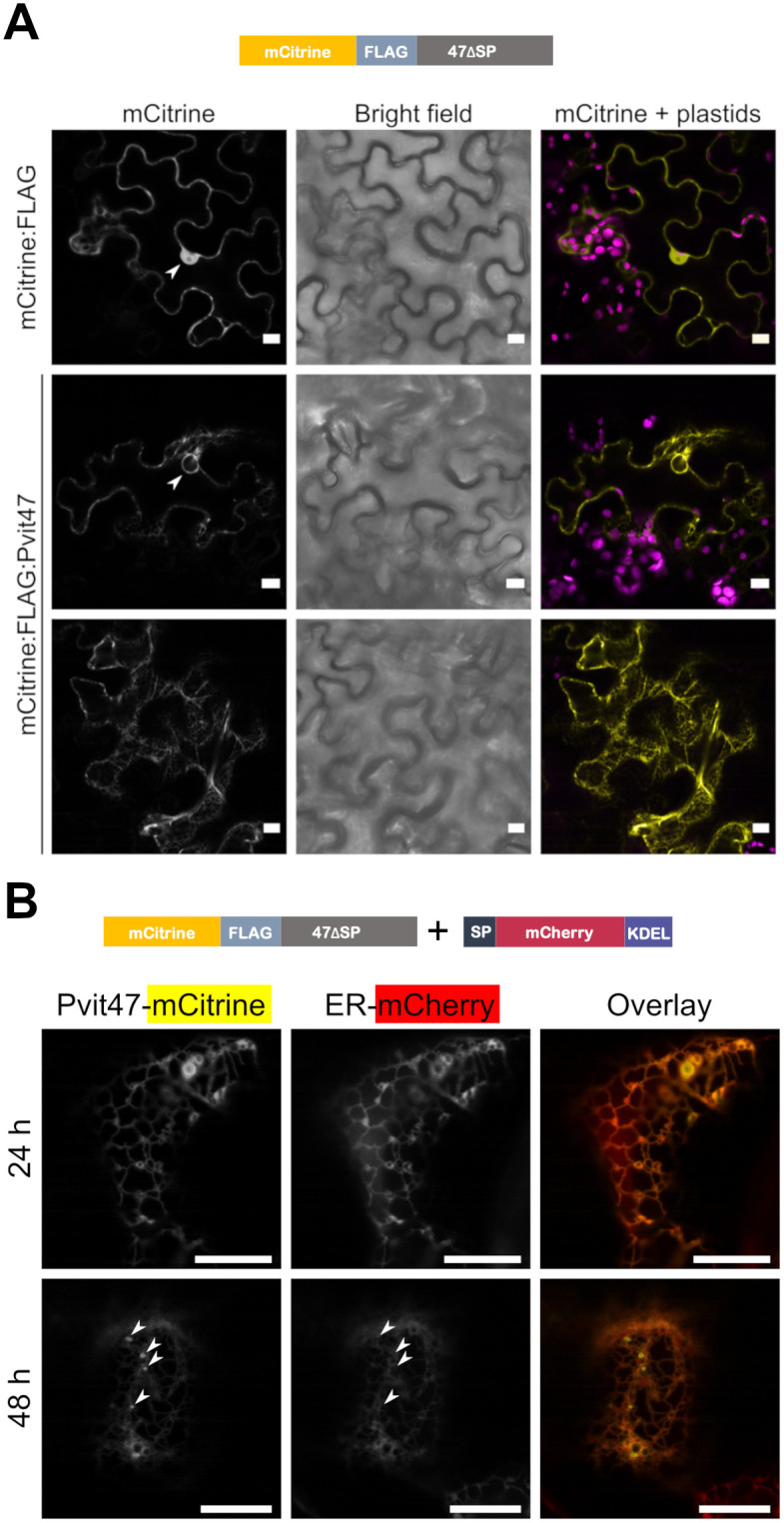
mCitrine-tagged Pvit47ΔSP localizes to the endoplasmic reticulum (ER). **(A)** Confocal microscopy images of *N*. *benthamiana* leaves transiently expressing mCitrine (top row) and mCitrine-tagged Pvit47ΔSP (two bottom rows). Arrowheads indicate nuclei. **(B)** Confocal microscopy images of *N*. *benthamiana* leaves transiently co-expressing mCitrine-tagged 47ΔSP and an ER-targeted version of mCherry. Arrowheads indicate punctate structures that occur at 48 hours post-agroinfiltration (hpa) in mCitrine-tagged Pvit47ΔSP but not in the ER marker. Images taken at 24- and 48-hpa (top and bottom respectively). KDEL is a four-amino acid ER retention motif. SP: signal peptide. Bars = 10 μm. The experiment was repeated once with the same results (S3 Fig).

To study the subcellular localization of Pvit47, we generated an mCitrine-tagged version of this protein without its signal peptide (Pvit47ΔSP). *Agrobacterium*-mediated transient expression of Pvit47 in *N*. *benthamiana* followed by confocal microscopy resulted in a localization pattern resembling the endoplasmic reticulum (ER, Fig 2A). Co-expression of the mCitrine-tagged version of Pvit47ΔSP with an ER-targeted version of mCherry resulted in both proteins showing the same localization pattern, confirming that mCitrine-Pvit47ΔSP is localized to the ER (Fig 2B, S3 Fig).

### Pvit47 expression in *N*. *benthamiana* reduces *Phytophthora parasitica* infection

**Fig 3:**
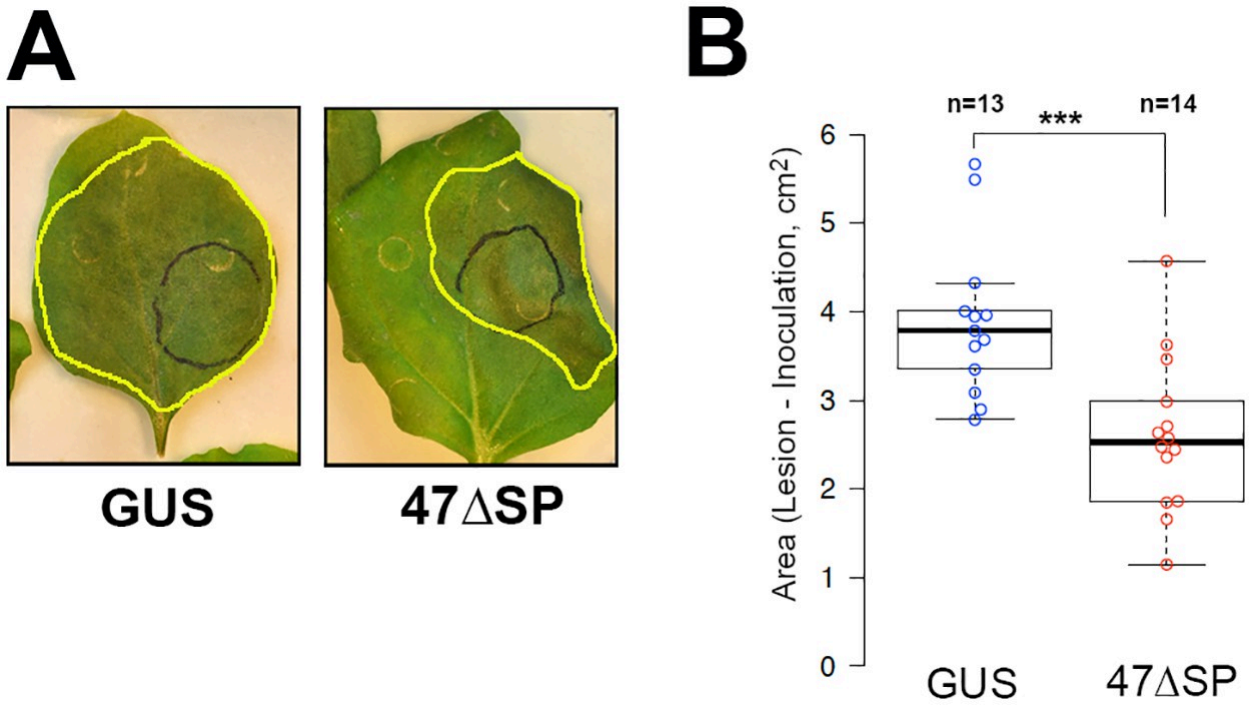
Pvit47 expression in *N*. *benthamiana* leaves reduces *P*. *parasitica* lesion size. Full *N*. *benthamiana* leaves were infiltrated with *Agrobacterium* strains carrying constructs for GUS (negative control) or Pvit47ΔSP (47ΔSP) and 2 days later *P*. *parasitica* was inoculated by infiltration of a spore suspension. **(A)** Representative images of leaves at 3 days post-inoculation (dpi). *P*. *parasitica* lesions are outlined in yellow and black circles indicate the area infiltrated with *P*. *parasitica* spores. **(B)** Quantification of lesion size at 3 dpi as necrotic area, excluding the inoculated area. The experiment was repeated a second time with similar results (S4 Fig). Asterisks show statistical significance of p<0.001 (***) in a two-tailed T-test for mean comparison.

In the context of biotrophic plant microbe interactions, cell death often results in arrest of pathogen growth. To study the effect of Pvit47ΔSP expression on pathogen infection, we performed *Agrobacterium*-mediated transient expression of Pvit47ΔSP in *N*. *benthamiana* leaves and two days later we inoculated the leaves with *P*. *parasitica*. Leaves infiltrated with *Agrobacterium* carrying a construct leading to the expression of GUS were used as a control. *Agrobacterium* carrying the Pvit47ΔSP and GUS constructs were infiltrated at a OD_600_ = 0.4 and *P*. *parasitica* was inoculated by infiltration of a spore suspension as previously reported [38]. Pathogen growth was scored three days post-inoculation (dpi) by measuring the necrotic area (excluding the infiltrated patch). *P*. *parasitica* lesion size was reduced in leaves agroinfiltrated with Pvit47ΔSP compared to leaves infiltrated with GUS (Fig 3 and S4 Fig).

### Pvit47 expression in *N*. *benthamiana* strongly reduces *Botrytis cinerea* infection

**Fig 4:**
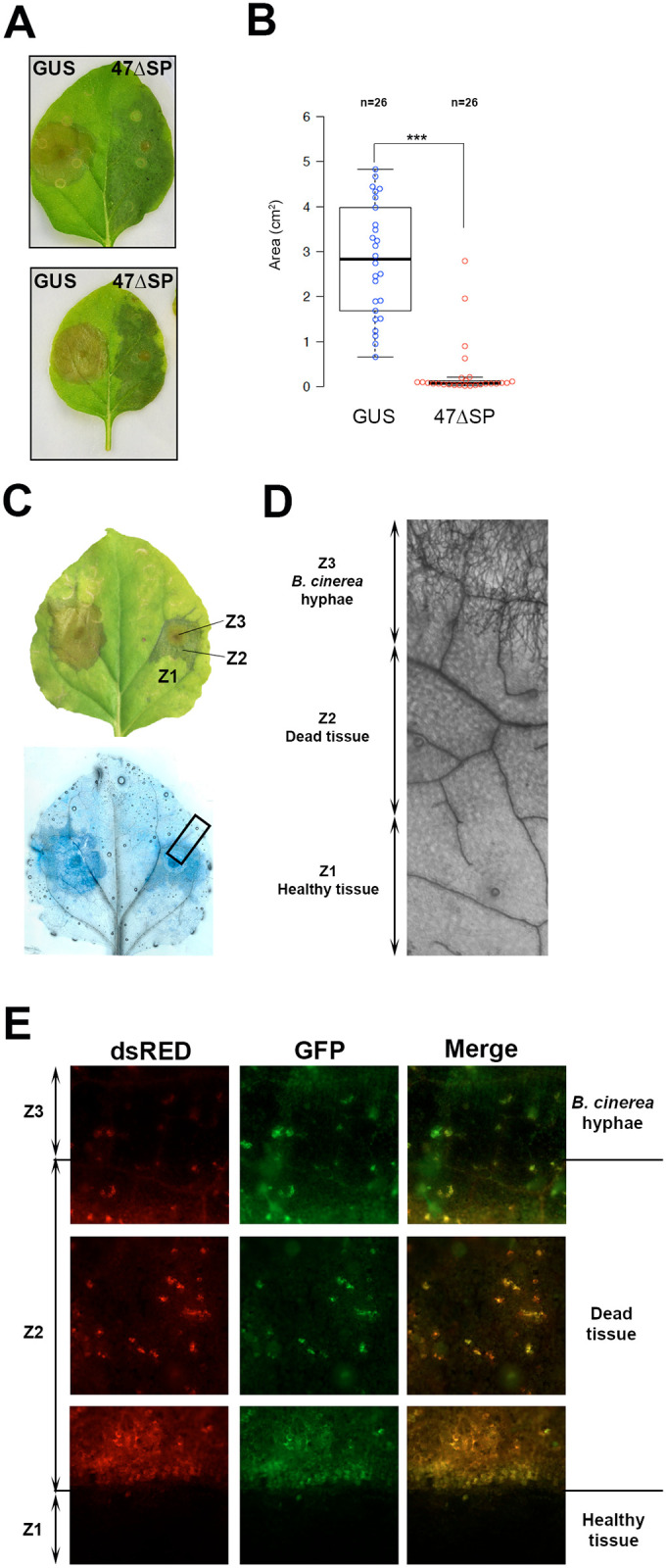
Pvit47 expression in *N*. *benthamiana* hinders *B*. *cinerea* infection. The two halves of *N*. *benthamiana* leaves were infiltrated with *A*. *tumefaciens* strains carrying constructs for GUS (negative control) or Pvit47ΔSP (47ΔSP) and 2 days later *B*. *cinerea* was inoculated by depositing a drop of a spore suspension. **(A)** Representative images of leaves at 4 dpi. **(B)** Quantification of lesion size at 4 dpi as necrotic area. Results pooled from three independent experiments. (***) indicates statistical significance at p<0.001 in a two-tailed T-test for mean comparison. **(C)** Daylight image and Trypan-blue staining of a representative leaf showing the different tissues observed in the half-leaves expressing 47ΔSP. Tissue zones are arbitrarily named Z1, Z2 and Z3 for further reference. **(D)** Magnification of the inset in C, showing the three types of tissues. **(E)** Fluorescence microscopy images following infection of a *N*. *benthamiana* leaf expressing 47ΔSP with a *B*. *cinerea* strain expressing GFP. Images are taken inside Z2 (middle) and at the Z1-Z2 and Z2-Z3 boundaries (top and bottom, respectively). Autofluorescence caused by cell death is scored in the dsRED and GFP channels. In the merged images, green reveals *B*. *cinerea* hyphae while orange exposes cell death.

We used the fungus *Botrytis cinerea* to study the effect of Pvit47ΔSP expression on a different pathogen. *Agrobacterium* carrying Pvit47ΔSP and GUS constructs were infiltrated side-by-side in *N*. *benthamiana* leaves and two days later leaves were inoculated with a spore suspension of *B*. *cinerea*. Pathogen growth was scored at 4 dpi by measuring the lesion size. Leaf sides expressing Pvit47ΔSP showed a strong reduction of *B*. *cinerea* growth compared to the sides expressing GUS (Fig 4A and 4B). Intriguingly, following inoculation with *B*. *cinerea*, leaf sides expressing Pvit47ΔSP developed a dark patch whose size varied depending on the experiments and did not necessarily correspond to the agroinfiltrated area (Fig 4A and 4C).

To gain insight into the nature of the patch, infected leaves were stained with trypan blue. Three different zones could be observed upon staining: healthy unstained tissue, dark-stained fungal hyphae and a light-blue-stained area corresponding to the patch (Fig 4C and 4D), which suggested that the patch was experiencing cell death. To test this hypothesis, we performed fluorescence microscopy using a GFP-tagged strain of *B*. *cinerea*. Cell death-induced autofluorescence was scored by merging signals from the green (525 nm) and red (629 nm) channels, which allowed autofluorescence to be distinguished from GFP-derived fluorescence. Results confirmed that the patch corresponded to tissues undergoing cell death (Fig 4D).

In summary, expression of Pvit47ΔSP strongly reduced *B*. *cinerea* infection whilst inducing the collapse of plant tissues around the infected area.

### Pvit47 contains WY- and LWY-domains

**Fig 5:**
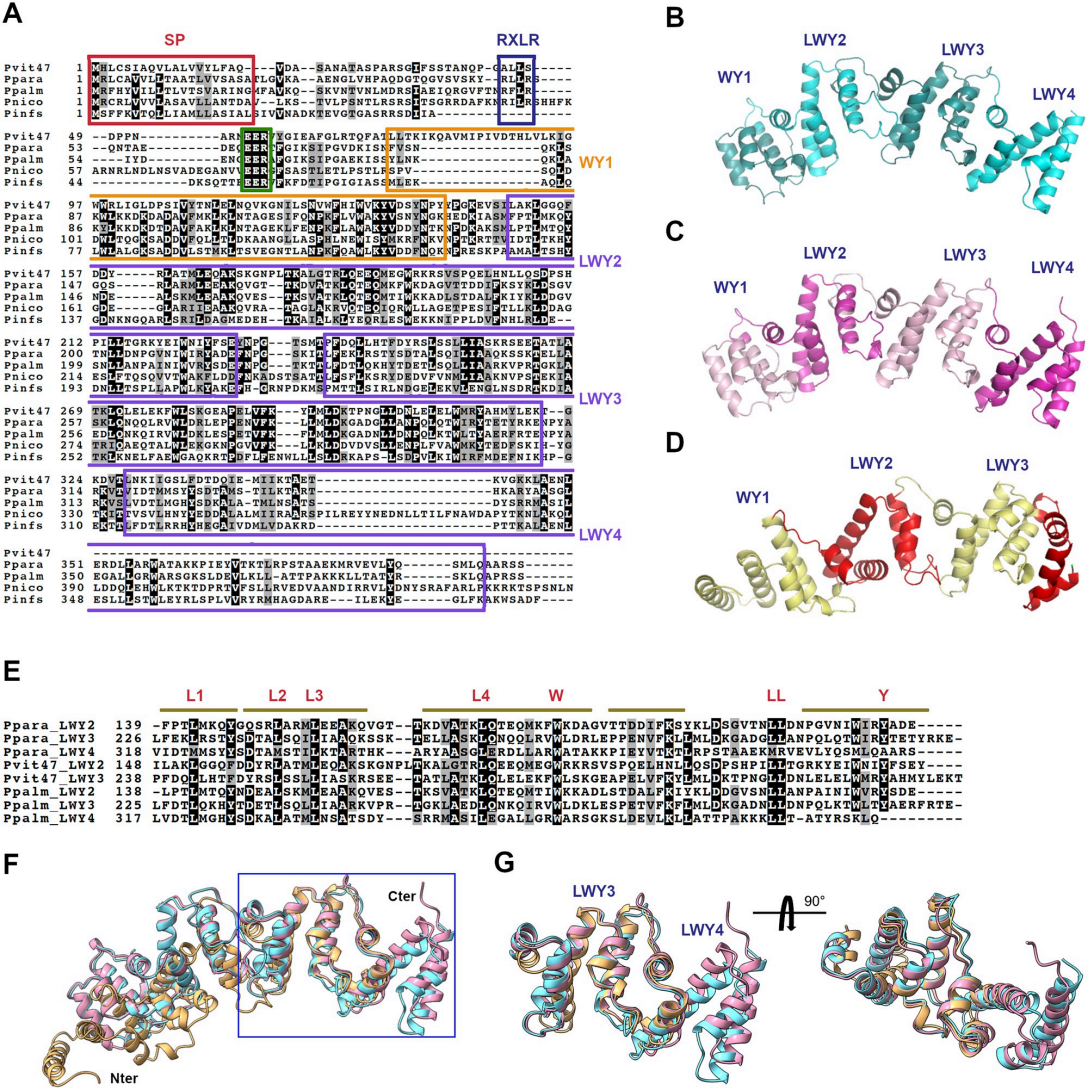
Pvit47 contains WY- and LWY-domains. **(A)** Alignment of Pvit47 from *Pl*. *viticola* with sequence-similar candidate effector proteins from *Phytophthora* species that infect *Nicotiana* species. Pinfs: *P*. *infestans*, Pnico: *P*. *nicotianae*, Ppara: *P*. *parasitica*, Ppalm: *P*. *palmivora*. Red box: signal peptide, blue box: RXLR motif, green box: EER motif; orange box: WY-domain, purple boxes: LWY-domain. **(B,C,D)** Predicted tertiary structures for candidate effector proteins from *P*. *parasitica* (B), *P*. *palmivora* (C) and Pvit47 (D). Predicted WY- and LWY-domains are indicated. N-terminal sequences up to the EER motif are not shown. **(E)** Alignment of LWY-domains from Pvit47 and candidate effector proteins from *P*. *parasitica* (Ppara) and *P*. *palmivora* (Ppalm). Bars represent α-helices on the Ppara sequence. Conserved residues defining the LWY-domain are show in red letters. **(F)** Superimposition of the predicted structures of Pvit47 (brown), Ppara (blue) and Ppalm (pink). N- and C-termini of the proteins are indicated. The region showing the best structural alignment between the three proteins is boxed. **(G)** Detail of the region boxed in F, comprising from LWY3 domain to the C-terminus, from two different angles. In (A) and (E), black background shows identity and grey background shows similarity (70% cutoff). Superimpositions in (F) and (G) were performed using the Pvit47 sequence as a template.

Pvit47 induces cell death in *Nicotiana* species but not in grapevine. Identifying proteins with similar cell death-inducing activity from *Nicotiana*-infecting oomycetes could help to understand the mechanisms underlying cell death induction. We searched the proteomes of the *Nicotiana*-infecting *Phytophthora infestans*, *Phytophthora nicotianae*, *Phytophthora palmivora* and *P*. *parasitica* for sequence-similar proteins and retrieved the closest proteins. All four proteins are predicted to be secreted and contain an EER motif and WY-domains, and the proteins from *P*. *palmivora*, *P*. *nicotianae* and *P*. *parasitica* have an RXLR motif (Fig 5A). Similarity of the retrieved sequences to Pvit47 is low, ranging from 35% to 44%. To gain insight into the possible structural similarity of these proteins, we performed *de novo* structural predictions using Alphafold2 for Pvit47 and two closest sequence-matching proteins from *P*. *parasitica* (Ppara) and *P*. *palmivora* (Ppalm). Models show overall high confidence, except for the first 80–90 residues (S5 Fig) and reveal that all three proteins contain WY- and LWY-domains (Fig 5B–5D). Sequence alignment of the predicted LWY-domains highlighted the conserved residues contributing to the fold (Fig 5E). Structural modelling of Pvit47, Ppara and Ppalm indicates that they share structural similarities. The structure models of Ppara and Ppalm consist of three LWY-domains, whilst the Pvit47 structure model contains two LWY-domains and what appears to be a truncated third domain. Superimposition of the predicted structures revealed that the Ppara an Ppalm structures align fully. By contrast, a comparison of Pvit47 with the Ppara and Ppalm structure models only showed good alignment in the C-terminal part of the protein (Fig 5F and 5G).

We next searched for similarity between Pvit47 and oomycete effectors described as inducing plant cell death. The closest proteins were PlAvh23 (22% identity, 38% similarity) from *Peronophythora litchi* and PaRXLR54 (23% identity, 38% similarity) from *Phytophthora agathidicida*. PaRXLR54 shows 40% identity and 58% similarity to Ppara, and its predicted structure also consists of a WY-domain and three LWY-domains (S6 Fig). Superimposition of the PaRXLR54 and Pvit47 predicted structures revealed the alignment of the N-terminal part of the protein, whereas superimposition of the PaRXLR54 and Ppara structures resulted in the alignment of the C-terminal part of the protein (S6 Fig).

To gain insight into the structural relationships between proteins, we quantified the level of similarity between LWY-domains by superimposing the predicted structures for individual LWY-domains and calculating the average per residue Root Mean Square Deviation (RMSD) for each structural alignment. Next, for each LWY-domain, we identified the structurally most similar LWY-domain from each other protein (lower average RMSD) (S7a Fig). For most LWY-domains, structural similarity was higher to LWY-domains from other proteins than to domains from the same protein. Co-linearity between proteins (i.e., for each LWY-domain from one protein, the most similar LWY-domain occupies the same position in the second protein) could be observed for three pairwise comparisons: Ppara-Ppalm, Ppara-PaRXLR54 and Pvit47-Ppalm. Furthermore, in all pairwise comparisons the best hits for LWY2 domains occupied the LWY2 position. It is worth noting the high level of structural similarity between the Ppalm and Ppara LWY-domains, which is in agreement with the superimposition of the two proteins (Fig 5F).

In parallel, we produced a sequence identity matrix and, for each LWY-domain, we identified the most similar LWY-domain from each other protein (S7b Fig). For all LWY-domains, similarity was higher to LWY-domains from other proteins than to domains from the same protein, and we could observe co-linearity in all pairwise comparisons between proteins except for Pvit47-PaRXLR54.

### A candidate RXLR effector from *Phytophthora parasitica* induces cell death in *Nicotiana* species

**Fig 6:**
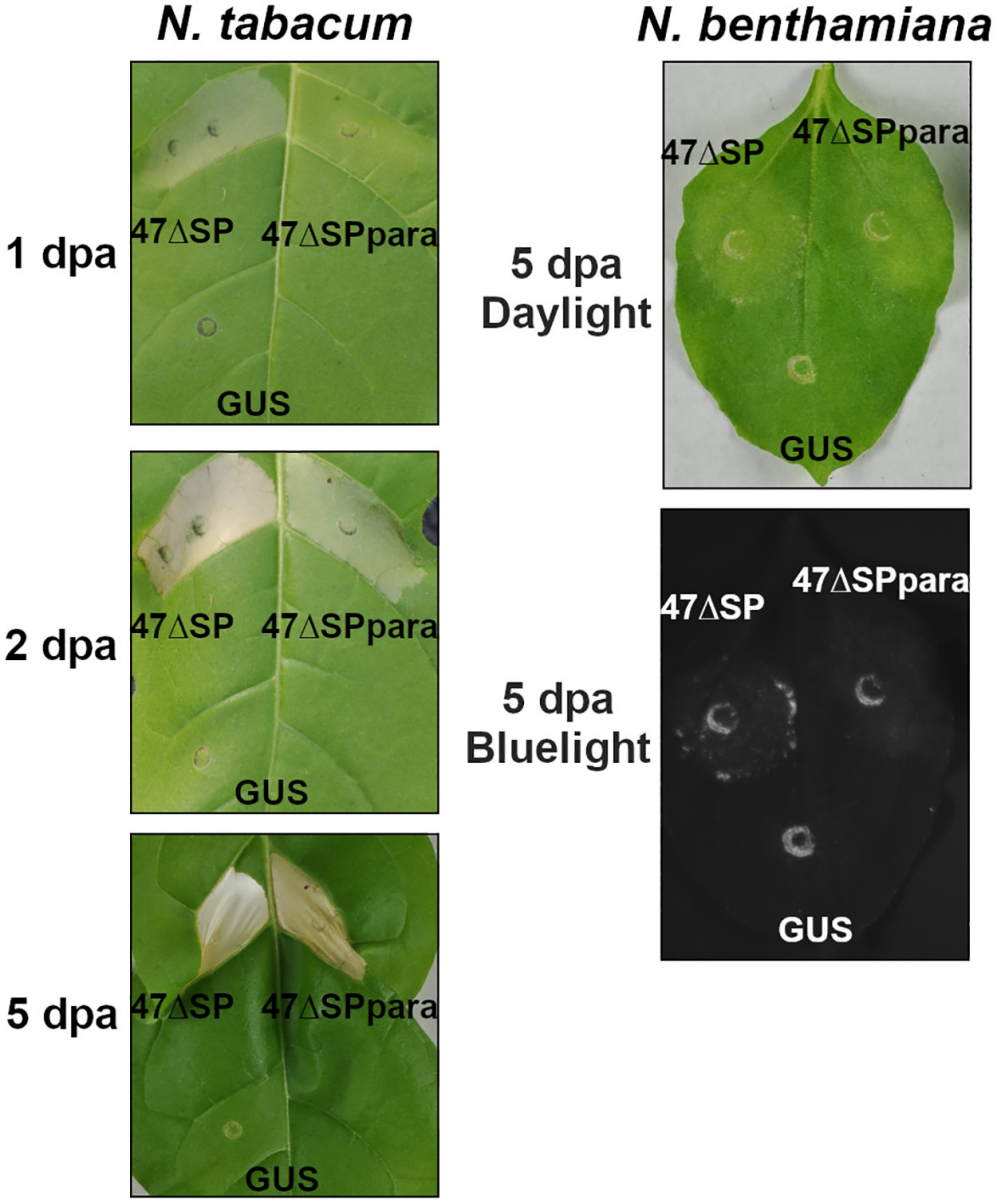
Ppar47 induces cell death in *Nicotiana* species. Cell death induction following *Agrobacterium*-mediated transient expression of Pvit47ΔSP (47ΔSP) and Ppar47ΔSP (47ΔSPpara) in leaves from *N*. *tabacum* (left) and *N*. *benthamiana* (right). *Agrobacterium*-mediated transient expression of GUS was used as negative control. Pictures were taken under daylight at 1, 2 and 5 days post-agroinfiltration (dpa) for *N*. *tabacum* and under daylight and blue light at 5 dpa for *N*. *benthamiana*. Results are representative of three independent experiments.

We cloned the coding sequence from the *P*. *parasitica* protein closest in amino acid sequence to Pvit47, which we hereby name Ppar47. *Agrobacterium*-mediated transient expression of Ppar47 without its signal peptide (Ppar47ΔSP) in leaves from *N*. *benthamiana* and *N*. *tabacum* resulted in induction of cell death in both species (Fig 6). In *N*. *tabacum*, the onset of the response induced by Ppar47ΔSP was delayed by 1 day compared to Pvit47ΔSP. Our results show that two putative effector proteins from *P*. *viticola* and *P*. *parasitica*, showing 44% sequence similarity as well as some level of structural similarity, induce similar cell-death responses when constitutively expressed in *Nicotiana* species.

## Discussion

Here we reported that Pvit47, a candidate effector protein from *Plasmopara viticola*, induced cell death in *Nicotiana* species but not in *V*. *vinifera*, the pathogen’s host species. Pvit47 is unrelated in sequence to any previously described *Pl*. *viticola* cell death-inducing effector but highly conserved among European isolates of the pathogen and expressed in sporangia, germinated spores and upon infection. By performing transient expression of the Pvit47 protein lacking its signal peptide (Pvit47ΔSP) in *Nicotiana benthamiana* leaves, we showed that the protein localizes to the endoplasmic reticulum and that it reduces infection by *Phytophthora parasitica* and *Botrytis cinerea*. Next, we showed that the closest protein from *P*. *parasitica*, which was a candidate RXLR effector 44% similar to Pvit47 that we named Ppar47, also exerted cell death activity in *Nicotiana* species. Finally, structural predictions revealed that Pvit47 and Ppar47 both contained WY- and LWY-domains.

Pvit47 is localized to the endoplasmic reticulum (ER), which is not common for effectors from plant-pathogenic oomycetes but has been described for *Hyaloperonospora arabidopsisis* (9 out of 50 effectors tested, [39]), *Plasmopara halstedii* (4 out of 30 effectors tested, [40]) and *P*. *infestans* [41]. The Pvit47 protein sequence does not carry a recognizable ER retention signal nor a predicted transmembrane domain. In absence of those targeting signals, the ER localization of Pvit47 most likely arises from an interaction with an ER-associated protein or other ER-associated molecule, as it has been reported for the Pi03182 RXLR effector from *P*. *infestans* [41].

Our study does not permit conclusions as to the relevance of Pvit47’s association with the ER. Experiments involving *Agrobacterium*-mediated transient expression of Pvit47 were performed using a cytoplasmic GUS as control to account for the eventual effect of agroinfiltration in cell death induction or pathogen growth. Pvit47 mutants without cell death activity and/or ER association would represent suitable alternative controls but were beyond the scope of this study.

Transient expression of Pvit47ΔSP in *N*. *benthamiana* leaves reduced the infection by the hemibiotrophic pathogen *P*. *parasitica*, suggesting that Pvit47 induces immune responses. Since the pathogen was inoculated as a spore suspension two days after transient expression of Pvit47ΔSP, we were most likely observing the effect of Pvit47 expression on the biotrophic phase of infection. While no visible cell death was visible at the time of inoculation, microscopic cell death could have contributed to this pathogen arrest regardless of induction of other immune responses.

The reduction of *B*. *cinerea* growth following transient expression of Pvit47 was accompanied by the appearance of a dark patch surrounding the infected area, corresponding to dead tissue (Fig 4). *B*. *cinerea* has an initial biotrophic phase in its infection cycle [42]. Our current understanding of the *B*. *cinerea* infection cycle implies two different cell death types: a plant-induced, autophagic cell death, aiming to block biotrophic pathogen development and a pathogen-induced, apoptotic cell death, allowing pathogen necrotrophic development [42, 43]. Under normal *N*. *benthamiana* infection conditions, once the biotrophic phase manages to overcome the plant-induced cell death there is pathogen-induced cell death, transition to the necrotrophic phase and pathogen growth. In *N*. *benthamiana* leaves transiently expressing Pvit47, there is an initial development of fungal hyphae that is later blocked even though the pathogen is surrounded by dead tissue which could support necrotrophic colonization (Fig 4). This observation could be explained if Pvit47-induced responses are not strong enough to block the biotrophic development of *B*. *cinerea*, but, following the secretion of cell death-inducing molecules allowing the beginning of the necrotrophic phase, there is a synergistic effect on the Pvit47-induced response (as observed by the dark patch), intensifying it and resulting in blocking of pathogen development. Under this hypothesis, Pvit47-induced responses may include immune responses which impair pathogen growth.

The intensity of the cell death response induced by Pvit47 and Ppar47 was dependent on the *Nicotiana* species. Both proteins induced strong cell death in *N*. *tabacum*, visible as soon as 1 dpa, and a weak response in *N*. *benthamiana*, most of the times barely visible macroscopically. Similar observations have been reported for the cell death responses mediated by the N disease resistance protein upon recognition of the TMV P50 protein and the INF2B elicitin from *P*. *infestans* [44, 45]. N-mediated responses lead to resistance to TMV, and INF2B-mediated responses have been suggested to be involved in the resistance of *N*.*tabacum* to *P*. *infestans*; it is thus tempting to speculate that Pvit47-induced cell death may also induce immune responses. It could be argued that *N*. *benthamiana* its somehow hindered in its ability to mount a cell death response, but results reported with other cell-death inducers like INF1, BcNEP and Pv33 [16, 44, 46], which behave similarly in both species, argue against this possibility.

Structural predictions revealed that Pvit47 and Ppar47 carried one WY-domain, and two and three LWY-domains, respectively (Fig 5). PaRXLR54, a third candidate effector with cell death-inducing activity, presented the same structural organization, and its primary sequence was 58% similar to Ppar47 (S6 Fig). Analyses of structural and sequence similarity of LWY-domains showed that Pvit47 and Ppar47 have co-linearity of LWY-domains at the sequence level but not at the structural level (S7 Fig), making it difficult to draw any conclusion about their eventual functional similarity. Indeed, based on sequence co-linearity it is tempting to speculate that both proteins may have similar functions, but, because function is based on structure, the lack of structural co-linearity prevents us from advancing such a hypothesis. The comparison between Pvit47 and PaRXLR54 did not provide much information regarding functional similarity, the only common point being the structural similarity between the LWY2s from both proteins. Interestingly, Ppar47 and PaRXLR54 showed co-linearity at the sequence and structural level (S7 Fig), so it is tempting to speculate that both proteins may be functional homologues. Further research will be required to know if cell death induction is the real function of the proteins or it is rather a proxy for their virulence activities due to overexpression.

In summary, here we reported two related candidate effector proteins from *Pl*. *viticola* and *P*. *parasitica* that trigger cell death in *Nicotiana* species when they are expressed inside plant cells. Although both oomycete species have different life styles and host ranges, recent phylogenetic analysis revealed a close relationship between *Plasmopara* species (*Pl*. *halstedii* and *Pl*. *viticola*) and several *Phytophthora* species, including *P*. *parasitica* [47–49]. This phylogenetic proximity could partly explain the fact that both candidate effector proteins trigger similar responses. The increasing number of oomycete effectors with assigned cell death activity when expressed alone in *N*. *benthamiana* may support a relevance in the infection biology of obligate biotrophic oomycetes. Less likely, cell death induction may be the consequence of these effectors all activating R protein-mediated responses. During an infection, other effectors could counteract specific effector cell death activities, alleviating a negative impact on pathogen development. Finally, it always remains possible that cell death induction may reflect the limitations of their overexpression in *N*. *benthamiana* as a system for the functional analysis of effector proteins.

## Materials and methods

### Plant and pathogen materials

*Vitis vinifera* Syrah was grown on soil from green cuttings in a greenhouse at 22°C-19°C (day/night) and with a photoperiod of 16h-8h (light/dark). New cuttings were produced every 3 months.

*Nicotiana benthamiana*, *N*. *tabacum* and *N*. *occidentalis* were grown on soil in a greenhouse at 28°C-18°C (max/min) and with a photoperiod of 14h/10h (light/dark; 10 klx min).

*Plasmopara viticola* isolate Pv221 was maintained in detached leaves of *V*. *vinifera* Muscat Ottonel. Methods for obtaining infected tissues and germinated spores have been described elsewhere [50].

*Phytophthora parasitica* strain 329 [38] was maintained in Malt Agar media at 24°C in darkness. Mycelium was transferred to fresh media every 3 week.

*Botrytis cinerea* strains BMM [51] and B05-10-GFP (B05.10 strain expressing GFP, kindly provided by Muriel Viaud at INRAE Versailles) were maintained in 5% clarified V8-agar media and transferred to fresh media every 2 weeks for a maximum of 4 transfers. New cultures were prepared from spores as described below.

### Sequence analysis

Search for *Phytophthora spp*. proteins similar to Pvit47 was performed by BlastP against the nr database at NCBI, limited to the taxon *oomycetes*. Accession numbers for the *Phytophthora spp*. proteins are: XP_002895862 (*P*. *infestans*), KUF89403 (*P*. *nicotianae*), ETI39065 (*P*. *parasitica*) and POM79043 (*P*. *palmivora*). Alignments were performed with ClustalW and displayed with Boxshade. Signal peptides were predicted with SignalPv5.0 [52]. LWY-domain identity matrix was done with ClustalW. Primary sequence from LWY-domains was extracted from structural predictions.

### Structural predictions

Structural predictions were performed using Alphafold2 [53] implemented at ColabFold [54] using default settings. Visualization and superimposition of predicted structures was performed on UCSF Chimera X 1.1.1 [55]. Root Mean Square Deviation (RMSD) calculations were carried out using UCSF ChimeraX software (https://www.cgl.ucsf.edu/chimerax/).

### Plasmid constructs

The coding sequences of Pvit47 and Ppar47 lacking their predicted signal peptides (Pvit47ΔSP and Ppar47ΔSP) were amplified by PCR with Phusion polymerase (NEB) from genomic DNA of *Pl*. *viticola* and *P*. *parasitica*, respectively, using primers containing restriction sites, digested (NEB restriction enzymes) and cloned directionally into plasmid pBIN61. Genomic DNA from *P*. *parasitica* strain 329 was extracted from mycelium using the Qiagen DNeasy Plant Mini kit. Genomic DNA isolation from *P*. *viticola* isolate Pv221 was performed using the same kit with the modifications described in [16]. Identity of the clones was confirmed by sequencing. Primers used for cloning are listed in S3 Table.

For mCitrine fusions, overlapping amplicons corresponding to mCitrine:FLAG and FLAG:Pvit47 were PCR-amplified with Phusion polymerase (NEB), separated by agarose gel electrophoresis and purified using the Qiagen MinElute Gel Extraction Kit. The final amplicon was obtained by overlap extension PCR using Phusion polymerase and a 1:10th dilution of the purified amplicons and subsequently cloned into a modified pUB-Dest vector as previously described [16].

Other constructs used in this study have been described elsewhere: 33ΔSP [16], mCherry-KDEL [56], GUS and GFP [57].

### *Agrobacterium*-mediated transient expression

*Agrobacterium*-mediated transient expression was performed as described in [58] for *Nicotiana spp*. and [16] for grapevine. In brief, for *Nicotiana spp*., *Agrobacterium* cultures were grown for 2 days at 28°C in 5 mL of L medium containing kanamycin (50 μg/mL) and tetracycline (2.5 μg/mL). Bacterial suspensions were centrifuged and the pellets were resuspended in a solution containing 10 mM MES, 10 mM MgCl_2_ and 150 μM acetosyringone. After 2–3 hours of incubation at room temperature, bacterial suspensions were infiltrated at an optical density at 600 nm (OD_600_) of 0.2 using a needleless syringe. For grapevine, bacterial cultures were grown for two days as described above. Then, 1 mL of the bacterial suspension was used to inoculate 5 mL of L medium containing kanamycin (50 μg/mL), tetracycline (2.5 μg/mL), 10 mM MES and 150 μM acetosyringone. Cultures were incubated in the same conditions for one day, centrifuged, resuspended in 10 mM MES, 10 mM MgCl_2_, 150 μM acetosyringone and 2% sucrose and incubated at room temperature for 2–3 hours. Infiltrations were performed on leaf discs by immerging the discs for 10 minutes in the bacterial solution (OD_600_ = 0.4) supplemented with 0.3% Silwet L-77.

### Semi-quantitative RT-PCR

RNA extraction, cDNA synthesis and PCR were done as in [58]. Each sample from infected tissues consisted of 4 leaf discs. Following RNA extraction, DNAse treatment was performed with the Invitrogen-Turbo DNA free kit, and first strand cDNA was synthetized using the RevertAid First Strand cDNA synthesis kit (Thermo Scientific). PCR amplifications consisted of 25 cycles of 20 s at 94°C, 20 s at 58°C and 60 s at 72°C, followed by a final extension step of 10 min at 72°C for *VvACT* and 30 cycles for *VvHSR*, *PvACT* and *Pvit47*. Primers are listed in S3 Table. Original images of the gels presented in Fig 1 and S2 Fig are shown in S1 Raw images.

### Pathogen inoculation

#### Phytophthora parasitica

Four plugs of 2-week-old mycelium grown in Malt-Agar media were transferred to Petri dishes containing 5% clarified V8-Agar media and dishes were incubated for 7 days at 24°C under continuous light. The resulting mycelium was recovered, cut into pieces, transferred to Petri dishes containing sterile distilled water with 2% agar and incubated at 24°C under continuous light for 4 days. To release the zoospores, dishes were incubated at 4°C for 1 hour, then 10 mL of sterile distilled water was added and the dishes were incubated at 37°C for 30 minutes. The water was recovered and zoospore number was measured using a Malassez cell-counting chamber. Spore concentration was adjusted at 10^3^ zoospores/mL and roughly 50 μl of zoospore suspension was infiltrated in the abaxial side of leaves of *N*. *benthamiana* plants. Leaves were detached, the infiltrated area was marked and leaves were placed abaxial side up in 90 mm Petri dishes containing wet filter paper (4 mL H_2_O). Dishes were sealed and incubated at 24°C with a 14h/10h (light/dark) photoperiod. Symptoms were scored by taking pictures at 3 days post-inoculation (dpi) and measuring the necrotic area with ImageJ using the *Freehand selection* and *Measure* tools.

#### Botrytis cinerea

Mycelium was grown on clarified V8-Agar on 90 mm Petri dishes. Spores were prepared by adding 5 mL of distilled sterile water and scraping the mycelium with a bacterial spreader. The suspension was collected, filtered through mesh and centrifuged at 250 g for 5 minutes. The pellet was then resuspended in 500 μl of distilled sterile water and the spore concentration was adjusted to 5x10^5^ sp/mL. Detached leaves from *N*. *benthamiana* were placed abaxial side up in 90 mm Petri dishes containing wet filter paper and a 5 μl drop of spore suspension was placed on the leaf surface. Dishes were sealed, covered with kitchen towel to dim the light and incubated at 22°C with a 16h/18h (light/dark) photoperiod. Symptoms were scored as for *P*. *parasitica*, with pictures taken at 4 dpi.

### Trypan-blue staining

Infiltrated *N*. *benthamiana* leaves were placed inside 50-mL Falcon tubes and incubated overnight in lactophenol-trypan blue solution (1 mL lactic acid, 1 mL glycerol, 10 mL phenol, 10 mg trypan blue, 10 mL distilled water). Samples were then boiled for 1 minute and distained by incubating twice for 60 min in chloral hydrate (2.5 g/mL). Leaves were transferred to glass plates and observed both macroscopically and using a stereomicroscope.

### Imaging

Visible light pictures of plant leaves were taken using a Nikon D5000 digital camera.

Epifluorescence microscopy images of *B*. *cinerea*-infected *N*. *benthamiana* leaves were obtained using a Zeiss Axio Imager M2 Microscope. Samples were excited at 470 nm and green and red fluorescence were observed using 525 nm and 629 nm filters, respectively.

Confocal laser scanning microscopy images of mCitrine-tagged Pvit47 were obtained with a Leica SP8 laser-scanning confocal microscope equipped with a 63× 1.2 numerical aperture (NA) objective (Leica, Wetzlar, Germany). A white-light laser was used for excitation at 514 nm for mCitrine and 580 nm for mCherry. Emission wavelengths were optimized with Leica Dye Assistant module (LAS X, Leica, Germany). Detection windows ranging from 525 nm to 555 nm and from 595 nm to 635 nm were used to detect mCitrine and mCherry, respectively.

## Supporting information

S1 Fig Protein sequence logo showing Pvit47 variability. Variability of Pvit47 in 18 European isolates of *Pl*. *viticola*. Signal peptide is coloured in blue and EER motif in green. Polymorphisms are shown in yellow and residues from the reference sequence in red. Conserved amino acids are shown in black. Sequences used to generate the logo are show in Dataset S1.
(TIF)

S2 Fig Pvit47 is expressed in spores and upon infection. Semi-quantitative RT-PCR of *Pvit47* expression in sporangia (Sp), germinated spores (Sg) and infected tissues at 0, 24, 48 and 72 hours post-inoculation (hpi). *V*. *vinifera Actin* (*VvActin*) expression is shown as equal loading of samples from infected tissues. *Pl*. *viticola Actin* (*PvActin*) expression reveals pathogen biomass and illustrates progression of infection. Amplicon sizes: *Pvit47* 1050 bp, *PvACT* 480 bp, *VvACT* 430 bp.
(TIF)

S3 Fig mCitrine-tagged Pvit47ΔSP localizes to the endoplasmic reticulum. Results of a second experiment for cellular localization of Pvit47. **(A)** Confocal microscopy images of *N*. *benthamiana* leaves transiently expressing mCitrine-tagged Pvit47ΔSP (47ΔSP). **(B)** Confocal microscopy images of *N*. *benthamiana* leaves transiently co-expressing mCitrine-tagged 47ΔSP and an ER-targeted version of mCherry. Bars = 15 μm. Images in B were obtained using a LSM700 confocal laser microscope (Carl Zeiss, Jena, Germany).
(TIF)

S4 Fig Pvit47 expression in *N*. *benthamiana* leaves reduces *P*. *parasitica* lesion size following spore inoculation. Results of a second experiment to study the effect of transient expression of Pvit47 on *P*. *parasitica* infection following inoculation as spore suspension. Methods and legends as described in the main text and Fig 3.
(TIF)

S5 Fig Confidence metrics of the predicted structures. Predicted template modelling (pTM) score, per-residue predicted local distance difference test (pLDDT) score of the five models proposed by Alphafold2 and predicted aligned error (pAE) score of the best ranked model are shown for each protein. pTM scores above 0.5 indicate confident predictions. pLDDT scores between 70 and 90 indicate good backbone prediction, while scores above 90 are associated to high accuracy prediction. For pAE scores, the lower the score the more confident the prediction.
(TIF)

S6 Fig Alignment of Pvit47 and cell death-inducing candidate RXLR effectors from other oomycetes. (A) Alignment of Pvit47, Ppar47, PlAvh23 from *Pe*. *litchi* and PaRXLR54 from *P*. *agathidicida*. Red box: signal peptide, blue box: RXLR, green box: EER motif; orange box: WY domain, purple boxes: LWY-domain. Black background shows identity, grey background shows similarity (70% cutoff). (B) Alignment of LWY-domains from Pvit47, Ppar47 and PaRXLR54. Conserved residues defining the LWY-domain are show in red letters. (C) Superimposition of the predicted structures of Pvit47 (brown), Pa RXLR54 (green). Superimposition done using Pvit47 as reference. (D) Superimposition of the predicted structures of Ppar47 (blue) and PaRXLR54 (green). Superimposition done using Ppar47 as reference.
(TIF)

S7 Fig Structural and sequence similarity of LWY-domains. (A) Average per residue Root Mean Square Deviation (RMSD) of structural alignments between LWY-domains of Pvit47, Ppara, Ppalm and PaRXLR54. Blue filling and white lettering indicate the structurally most similar LWY-domain for each domain in a pairwise protein comparison. (B) Sequence identity matrix of LWY-domains of Pvit47, Ppara, Ppalm and PaRXLR54. Blue filling and white lettering indicate the most sequence-similar LWY-domain for each domain in a pairwise protein comparison.
(TIF)

S1 Table Cell-death responses observed following *Agrobacterium*-mediated transient expression of Pvit47ΔSP in *N*. *benthamiana* leaves. Results from five independent experiments. Representative images for each class are presented in Fig 1A.
(PDF)

S2 Table European isolates of *Plasmopara viticola* used for the study of Pv47 variability leading to the logo presented in S1 Fig. Geographical coordinates for each collection point and resequencing information for the isolates is described in Dussert el al 2020 (https://doi.org/10.1016/j.cub.2020.07.057).
(PDF)

S3 Table Primers used in this study. (PDF)

S1 Dataset Protein sequences used to generate the WebLogo shown in S1 Fig. (TXT)

S1 Raw images Original images for gels presented in Fig 1 and S2 Fig. (PDF)
