# Supplementary figures and images for: Candidate effector proteins from the oomycetes Plasmopara viticola and Phytophthora parasitica share similar predicted structures and induce cell death in Nicotiana species
Source: PLoS One. 2022 Dec 2;17(12):e0278778. doi: 10.1371/journal.pone.0278778 (PMC9718384; doi:10.1371/journal.pone.0278778)

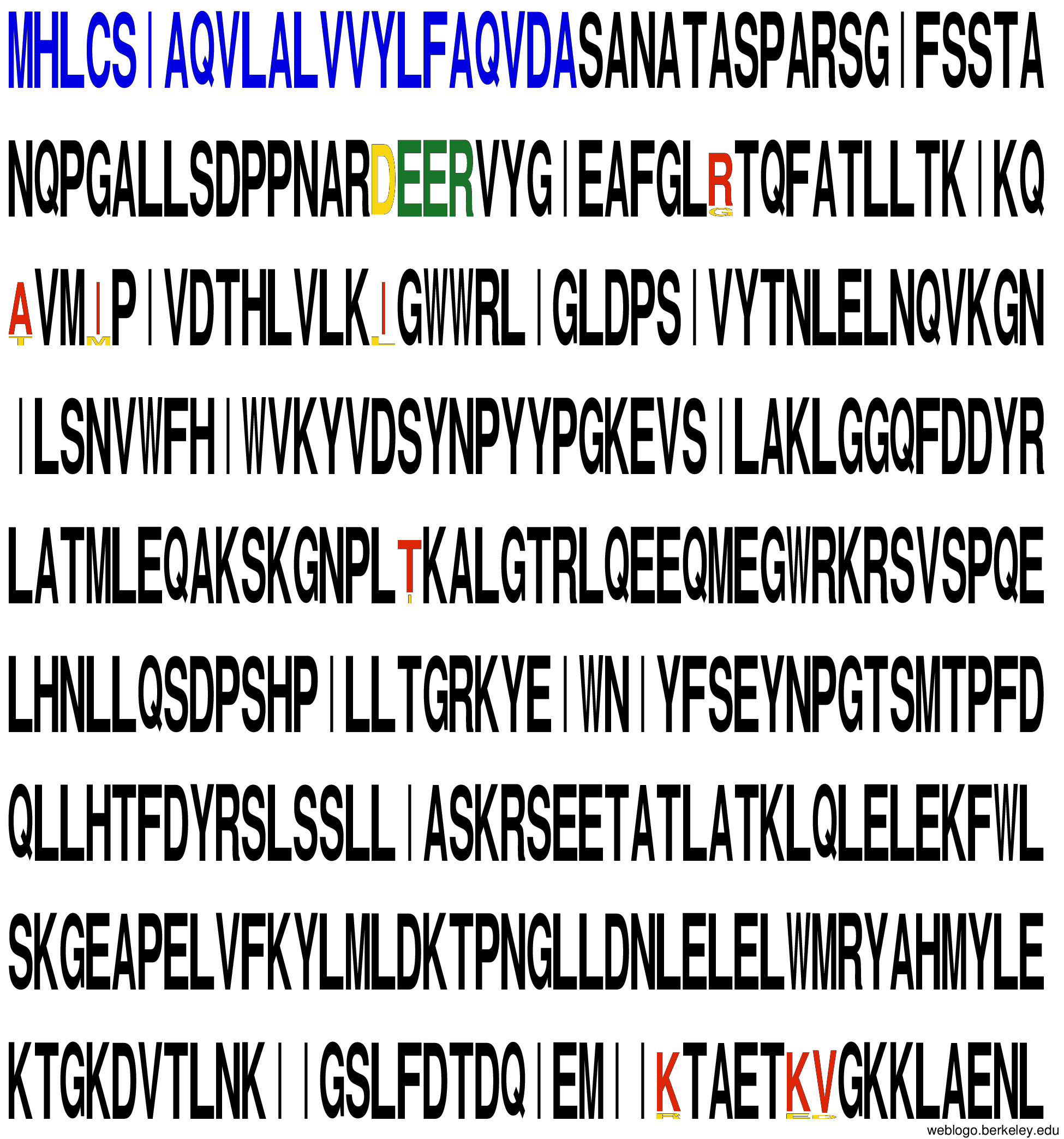

Supplement: S1 Fig — Variability of Pvit47 in 18 European isolates of Pl. viticola. Signal peptide is coloured in blue and EER motif in green. Polymorphisms are shown in yellow and residues from the reference sequence in red. Conserved amino acids are shown in black. Sequences used to generate the logo are show in Dataset S1. (TIF) [file pone.0278778.s001.tif]

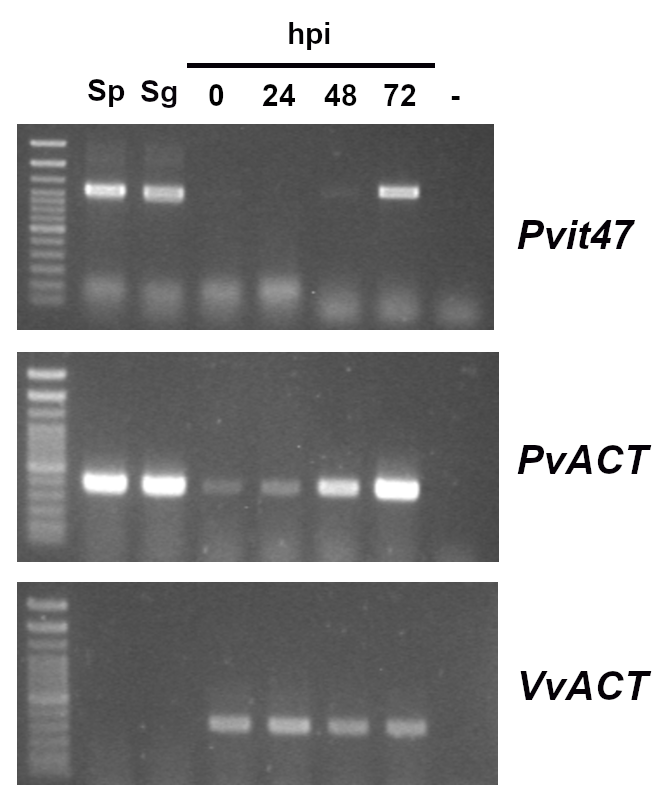

Supplement: S2 Fig — Semi-quantitative RT-PCR of Pvit47 expression in sporangia (Sp), germinated spores (Sg) and infected tissues at 0, 24, 48 and 72 hours post-inoculation (hpi). V. vinifera Actin (VvActin) expression is shown as equal loading of samples from infected tissues. Pl. viticola Actin (PvActin) expression reveals pathogen biomass and illustrates progression of infection. Amplicon sizes: Pvit47 1050 bp, PvACT 480 bp, VvACT 430 bp. (TIF) [file pone.0278778.s002.tif]

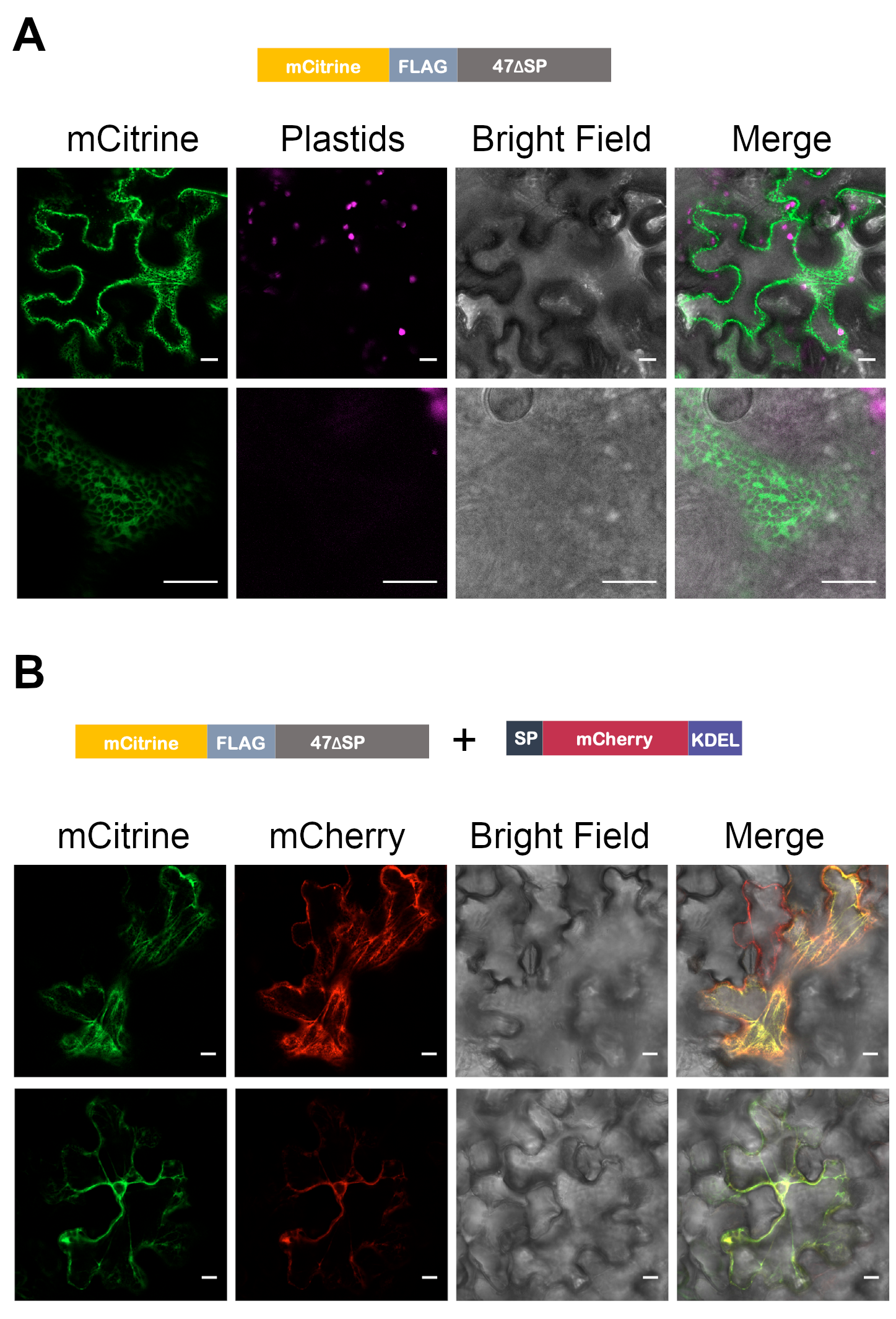

Supplement: S3 Fig — Results of a second experiment for cellular localization of Pvit47. (A) Confocal microscopy images of N. benthamiana leaves transiently expressing mCitrine-tagged Pvit47ΔSP (47ΔSP). (B) Confocal microscopy images of N. benthamiana leaves transiently co-expressing mCitrine-tagged 47ΔSP and an ER-targeted version of mCherry. Bars = 15 μm. Images in B were obtained using a LSM700 confocal laser microscope (Carl Zeiss, Jena, Germany). (TIF) [file pone.0278778.s003.tif]

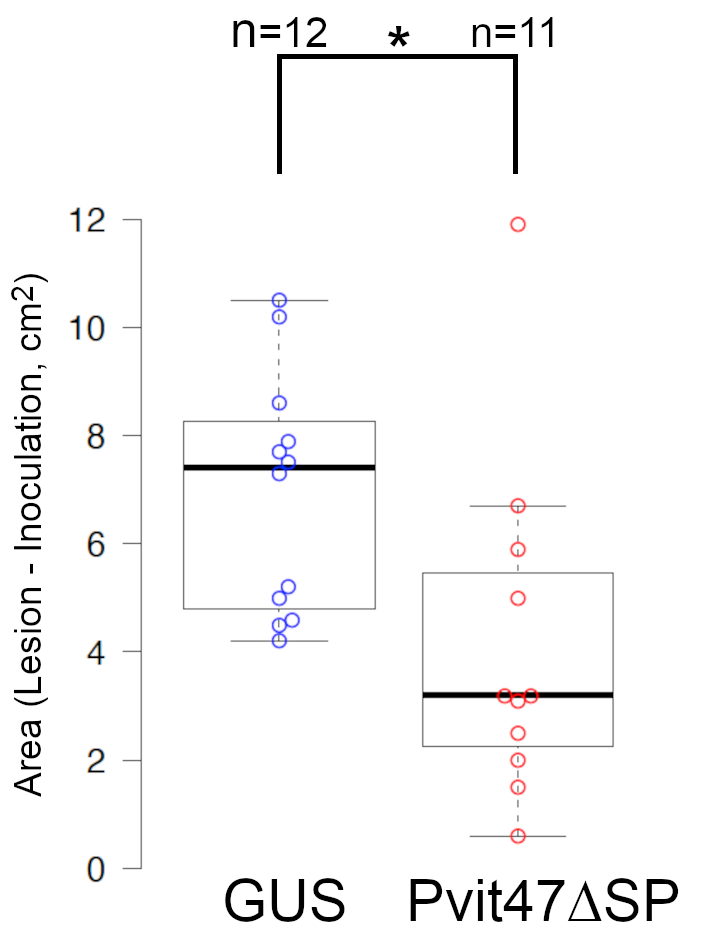

Supplement: S4 Fig — Results of a second experiment to study the effect of transient expression of Pvit47 on P. parasitica infection following inoculation as spore suspension. Methods and legends as described in the main text and Fig 3. (TIF) [file pone.0278778.s004.tif]

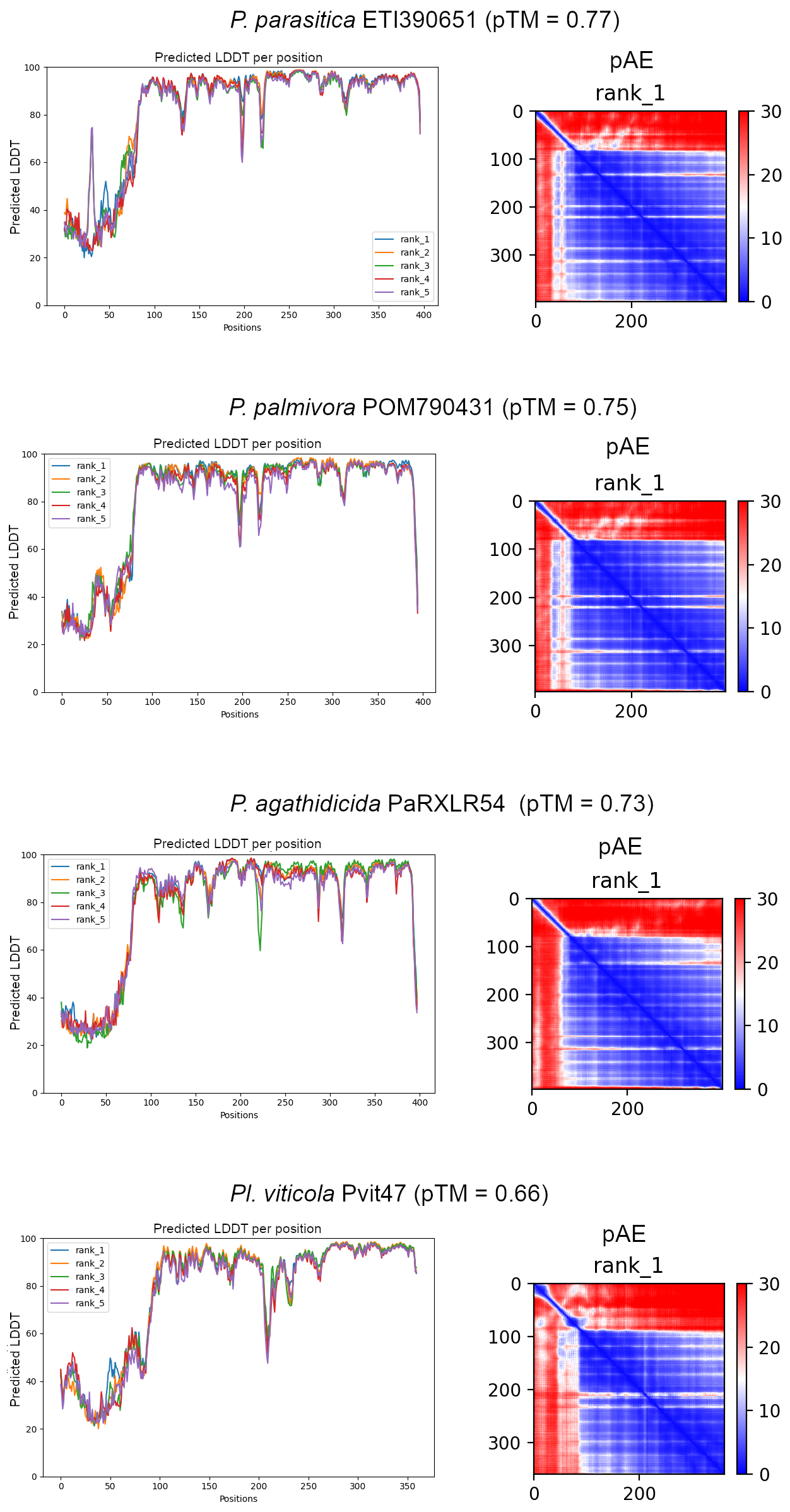

Supplement: S5 Fig — Predicted template modelling (pTM) score, per-residue predicted local distance difference test (pLDDT) score of the five models proposed by Alphafold2 and predicted aligned error (pAE) score of the best ranked model are shown for each protein. pTM scores above 0.5 indicate confident predictions. pLDDT scores between 70 and 90 indicate good backbone prediction, while scores above 90 are associated to high accuracy prediction. For pAE scores, the lower the score the more confident the prediction. (TIF) [file pone.0278778.s005.tif]

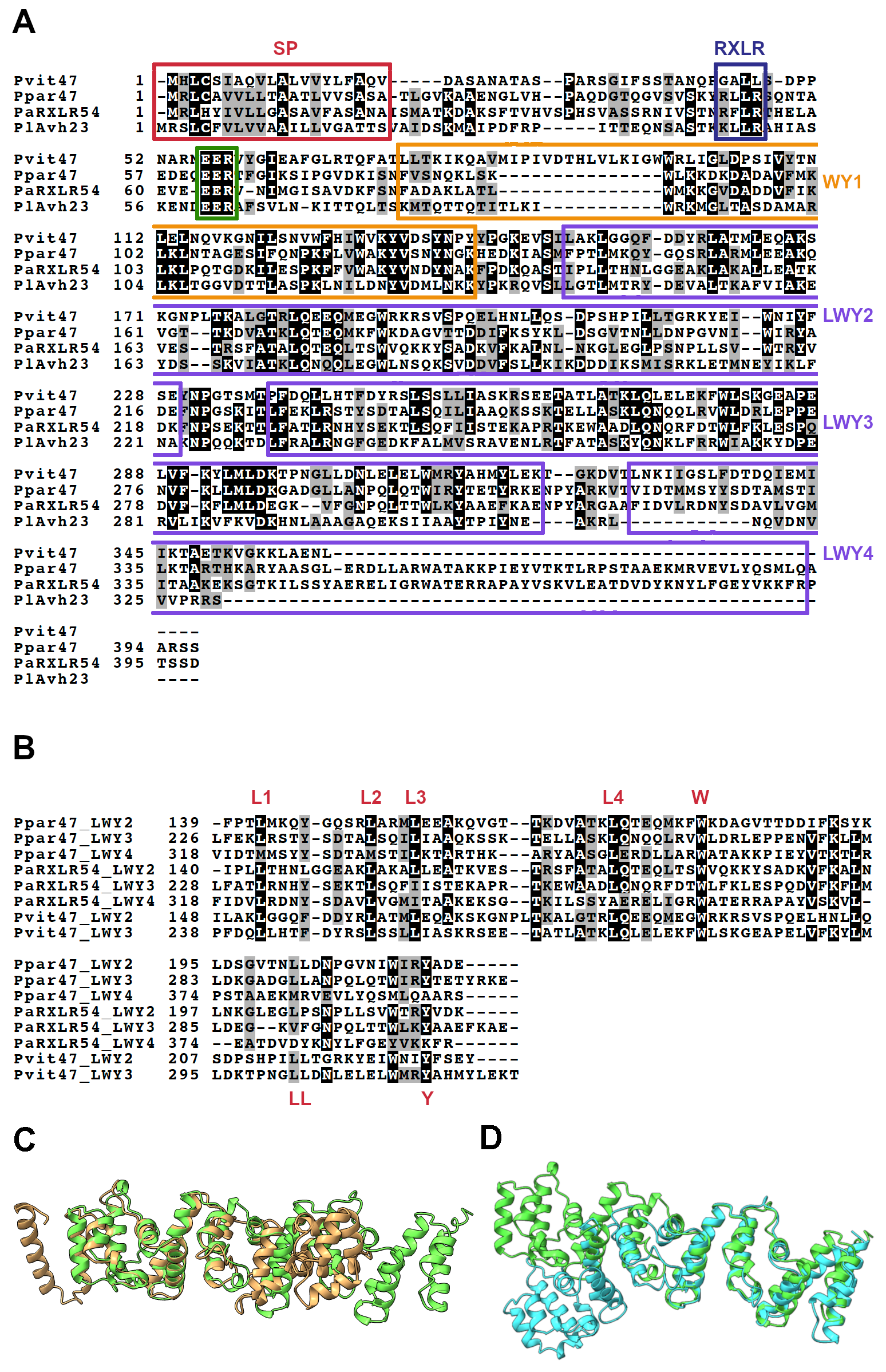

Supplement: S6 Fig — (A) Alignment of Pvit47, Ppar47, PlAvh23 from Pe. litchi and PaRXLR54 from P. agathidicida. Red box: signal peptide, blue box: RXLR, green box: EER motif; orange box: WY domain, purple boxes: LWY-domain. Black background shows identity, grey background shows similarity (70% cutoff). (B) Alignment of LWY-domains from Pvit47, Ppar47 and PaRXLR54. Conserved residues defining the LWY-domain are show in red letters. (C) Superimposition of the predicted structures of Pvit47 (brown), Pa RXLR54 (green). Superimposition done using Pvit47 as reference. (D) Superimposition of the predicted structures of Ppar47 (blue) and PaRXLR54 (green). Superimposition done using Ppar47 as reference. (TIF) [file pone.0278778.s006.tif]

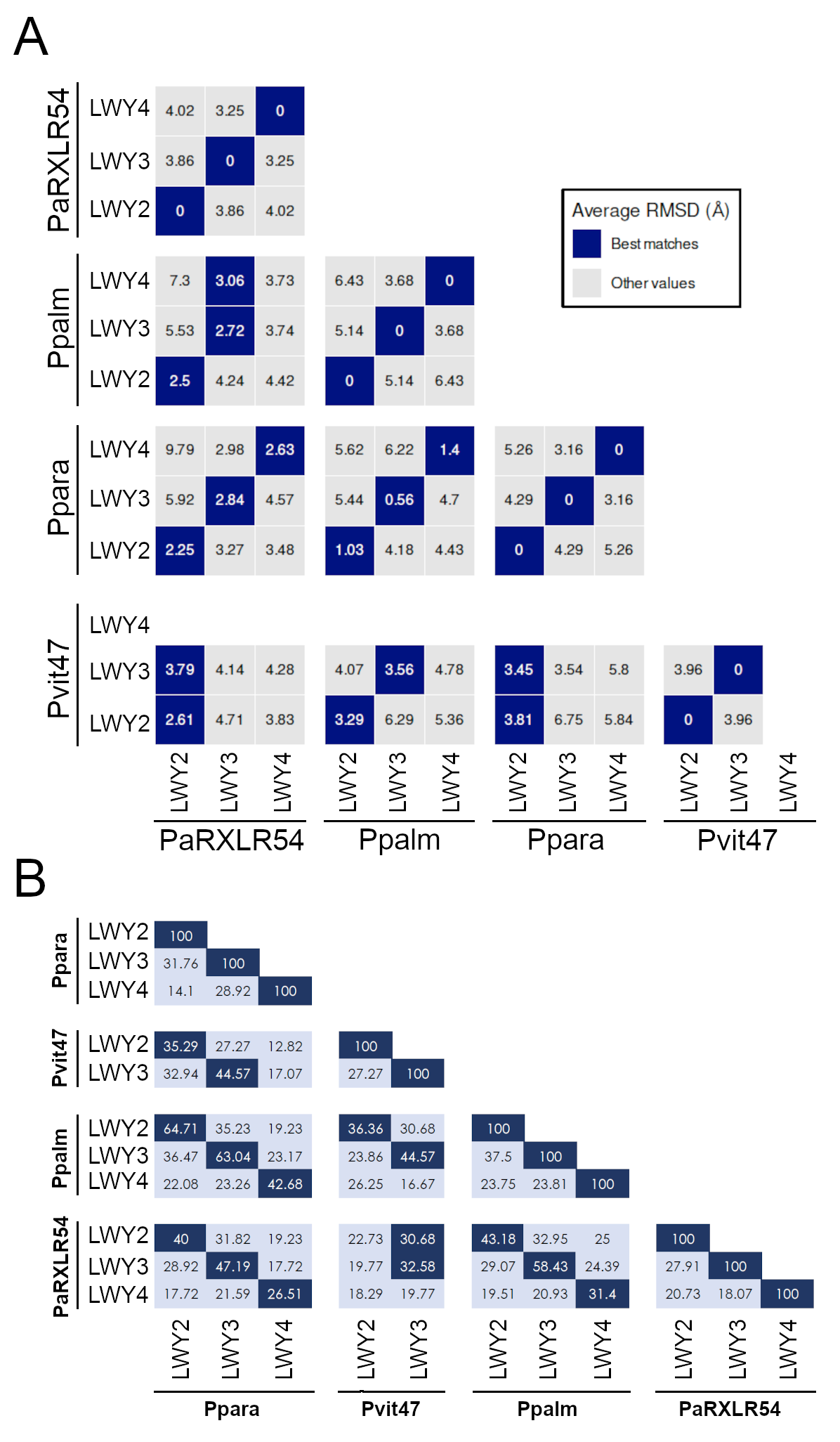

Supplement: S7 Fig — (A) Average per residue Root Mean Square Deviation (RMSD) of structural alignments between LWY-domains of Pvit47, Ppara, Ppalm and PaRXLR54. Blue filling and white lettering indicate the structurally most similar LWY-domain for each domain in a pairwise protein comparison. (B) Sequence identity matrix of LWY-domains of Pvit47, Ppara, Ppalm and PaRXLR54. Blue filling and white lettering indicate the most sequence-similar LWY-domain for each domain in a pairwise protein comparison. (TIF) [file pone.0278778.s007.tif]
