# Supplementary material for: Candidate effector proteins from the oomycetes Plasmopara viticola and Phytophthora parasitica share similar predicted structures and induce cell death in Nicotiana species
Source: PLoS One. 2022 Dec 2;17(12):e0278778. doi: 10.1371/journal.pone.0278778 (PMC9718384; doi:10.1371/journal.pone.0278778)
Supplement: S1 Table — Results from five independent experiments. Representative images for each class are presented in Fig 1A. (PDF) [file pone.0278778.s008.pdf]

S1 Table. Cell-death responses observed following *Agrobacterium*-mediated transient expression of Pvit47ΔSP in *N. benthamiana* leaves. Results from five independent experiments. Representative images for each class are presented in Figure 1A.

Experiment	Number of patches	Cell death response				
		Class I	Class II	Class III	Class IV	Class V
#1	4	0	0	2	2	0
#2	9	0	1	4	2	2
#3	8	1	5	2	0	0
#4	4	4	0	0	0	0
#5	8	1	5	2	0	0
**Total**	**33**	**6**	**11**	**10**	**4**	**2**

Class I: no macroscopic response.
Class II: yellowing/weak cell death with no tissue collapse.
Class III: tissue collapse patchy on less than 25% of the patch surface.
Class IV: tissue collapse patchy on 25%-75% of the patch surface.
Class V: tissue collapse uniform.
