# Supplementary material for: Candidate effector proteins from the oomycetes Plasmopara viticola and Phytophthora parasitica share similar predicted structures and induce cell death in Nicotiana species
Source: PLoS One. 2022 Dec 2;17(12):e0278778. doi: 10.1371/journal.pone.0278778 (PMC9718384; doi:10.1371/journal.pone.0278778)
Supplement: S2 Table — Geographical coordinates for each collection point and resequencing information for the isolates is described in Dussert el al 2020 (https://doi.org/10.1016/j.cub.2020.07.057). (PDF) [file pone.0278778.s009.pdf]

S2 Table. European isolates of *Plasmopara viticola* used for the study of Pv47 variability leading to the Logo presented in S1 Fig. Geographical coordinates for each collection point and resequencing information for the isolates is described in Dussert el al 2020. (https://doi.org/10.1016/j.cub.2020.07.057).

**Isolate**	**Host[a]**	**Origin**
PV13	Regent	Latresne, France
PV125	Regent	Pécs, Hungary
PV221	*V. vinifera*	Blanquefort, France
PV319	*V. vinifera*	Cote d'Or, France
PV321	*V. vinifera*	Kröv, Germany
PV330	Regent	Pfaffenweiler, Germany
PV334	*V. vinifera*	Ehrenkirchen, Germany
PV336	*V. vinifera*	Eger, Hungary
PV365	Bianca	Cugnasco, Switzerland
PV375	Johanniter	Cugnasco, Switzerland
PV392	*V. vinifera*	Cugnasco, Switzerland
PV1085	Chasselas	Chexbres, Switzerland
PV1102	Johanniter	Nyon, Switzerland
PV1174	Pinot Noir	Ihringen, Germany
PV1606	*V. vinifera*	Leytron, Switzerland
PV1614	Regent	Pully, Switzerland
PV1645	*V. vinifera*	Stäfa, Switzerland
PV1989	Gamay	Pouilly Le Monial, France

a: The name of the variety is stated when known; otherwise, the species name is used.
