# Supplementary material for: Candidate effector proteins from the oomycetes Plasmopara viticola and Phytophthora parasitica share similar predicted structures and induce cell death in Nicotiana species
Source: PLoS One. 2022 Dec 2;17(12):e0278778. doi: 10.1371/journal.pone.0278778 (PMC9718384; doi:10.1371/journal.pone.0278778)
Supplement: S3 Table — (PDF) [file pone.0278778.s010.pdf]

S3 Table. Primers used in this study.

Primers for cloning in binary plasmids (restriction sites underlined)

PvRXL47sp	5’ GCGCAATCTAGAATGTCTGCGAATGCGACTG
PvRXL47R	5’ GCTTACCCCGGGCTACAAATTTTCCGCCAAC
47SPparaF	5’ TAGTCGTCTAGAATGACATTAGGCGTGAAAGC
47SPparaR	5’ ACGTTGCCCGGGCTAGGAAGAGCGAGCCGCT

Primers for *P. viticola Actin*, *V. vinifera HSR* and *V. vinifera Actin* for RT-PCR

VvACT-F	5’ TACAATGAGCTTCGGGTTGC
VvACT-R	5’ GCTCTTTGCAGTTTCCAGCT
VvHSR-F	5’ GGACTACCGACATGCACCTG
VvHSR-R	5’ CCTGGACAATTCTGCCATCT
PvACT-F	5’ GTTCGAGACGTTCAACGTGC
PvACT-R	5’ CATGATGGTCTGGAACGTGC
