# Supplementary material for: Candidate effector proteins from the oomycetes Plasmopara viticola and Phytophthora parasitica share similar predicted structures and induce cell death in Nicotiana species
Source: PLoS One. 2022 Dec 2;17(12):e0278778. doi: 10.1371/journal.pone.0278778 (PMC9718384; doi:10.1371/journal.pone.0278778)
Supplement: S1 Raw images — (PDF) [file pone.0278778.s012.pdf]

Original images for gels shown in Figures 1D and S2.
Images taken with a Geldoc imaging system from Bio-Rad

Figure 1D

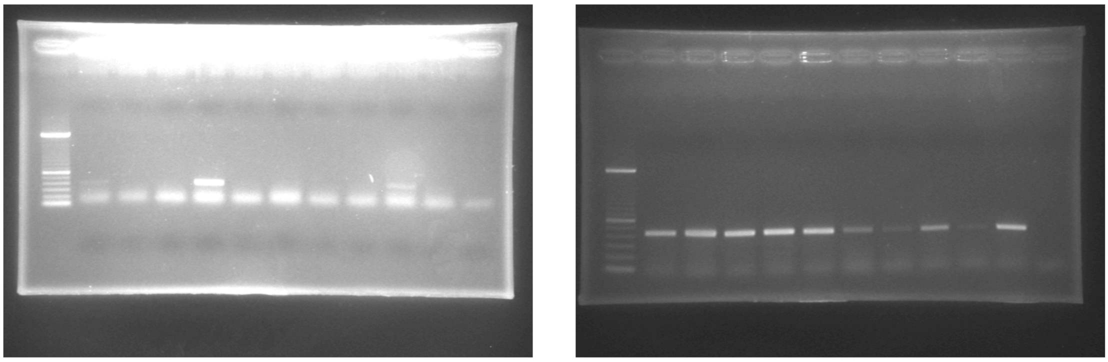

Figure 1D top pannel

Figure 1D bottom pannel

Figure S2

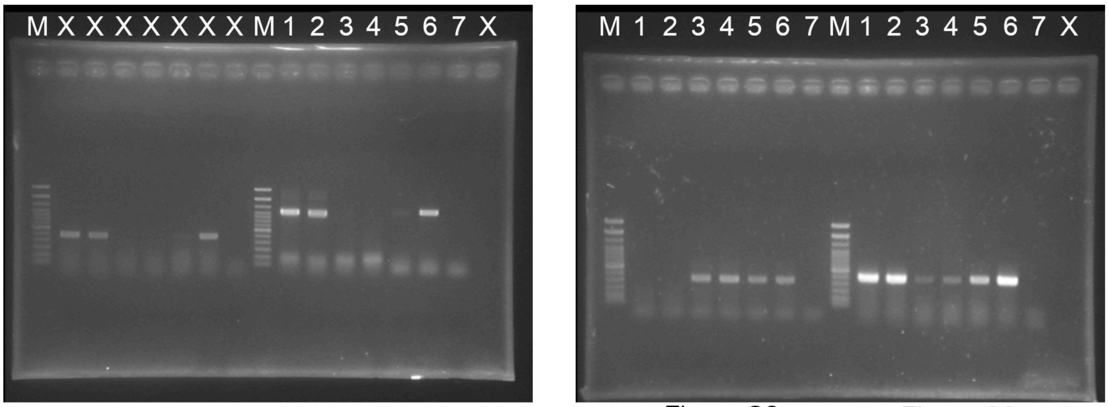

Figure S2
Top pannel

Figure S2
Bottom pannel

Figure S2
Middle pannel

M: Invitrogen 100 bp DNA ladder
